# Retroperitoneal Laparoscopic Partial Adrenalectomy in a Patient with Conn’s Syndrome

**DOI:** 10.5152/tud.2023.23102

**Published:** 2023-07-01

**Authors:** Syed Jamal Rizvi, Kamleshkumar Ramabhai Patel, Sreenivasan R. Prasad, Ketan Mehra

**Affiliations:** Department of Urology, Institute of Kidney Disease and Research Centre (IKDRC), Ahmedabad, India

## Objective

Laparoscopic adrenalectomy has the advantage of reduced blood loss, early convalescence, and shorter hospital stays. Retroperitoneal laparoscopic approach was first demonstrated by Mercan et al. in 1995 and is easier for adrenal pathologies. Adrenal-preserving surgeries may prevent adrenal insufficiency from developing over long time. There are many studies in the literature which have compared the appropriate approach and the extent of resection for the functional adrenal tumor. But it remains unclear which is the optimal option. Here we present a video of laparoscopic partial adrenalectomy by a retroperitoneal approach in a patient with primary hyperaldosteronism.

## Methods

This procedure was performed by laparoscopic retroperitoneal approach for a small right adrenal lesion, which was an aldosterone-secreting benign tumor diagnosed preoperatively. Initially after creating a retroperitoneal space with the help of a balloon, the procedure was carried out with 2 working ports apart from the camera port. Intraoperatively, a well-circumscribed 2 cm adrenal lesion was identified. A partial adrenalectomy was performed.

## Results

Total operating time was 1 hour 40 minutes. Estimated blood loss was 100 mL. There was no intraoperative or postoperative complication. In the postoperative period, the patient got relieved of the symptoms, and her antihypertensive drug requirements were reduced from 4 to a single drug. The patient was discharged on the third postoperative day.

## Conclusion

In summary, retroperitoneal laparoscopic partial adrenalectomy is an easy, fast, and organ-preserving approach that can be performed for unilateral hormone-secreting tumors.

Video see link: https://doi.org/10.5152/tud.2023.23102

### Ethics Committee Approval:

This study was approved by Ethics Committee of Gujarat University of Transplantation Sciences (GUTS) University).

### Informed Consent:

Verbal informed consent was obtained from the patient who agreed to take part in the study.

### Peer-review:

Externally peer-reviewed.

### Author Contributions:

Concept – S.R., K.M.; Design – S.R., K.M.; Supervision – S.R., K.P.; Resources – S.P., K.P.; Materials – K.M., K.P.; Data Collection and/or Processing – K.M., S.P.; Analysis and/or Interpretation – S.R., K.M.; Literature Search – K.M., S.P.; Writing – K.M., S.P.; Critical Review – S.R., K.M.

### Declaration of Interests:

The authors have no conflict of interest to declare.

### Funding:

The authors declared that this study has received no financial support.

